# The Clinical Role of Angiopoietin-Like Protein 3 in Evaluating Coronary Artery Disease in Patients with Obstructive Sleep Apnea

**DOI:** 10.1007/s10557-020-06991-1

**Published:** 2020-05-21

**Authors:** Juan Li, Yunyun Yang, Xiaolu Jiao, Huahui Yu, Yunhui Du, Ming Zhang, Chaowei Hu, Yongxiang Wei, Yanwen Qin

**Affiliations:** 1 Key Laboratory of Upper Airway Dysfunction-related Cardiovascular Diseases, Beijing An Zhen Hospital, Capital Medical University, Beijing Institute of Heart, Lung, and Blood Vessel Diseases, Beijing, 100029 China; 2 Key Laboratory of Remodeling-related Cardiovascular Diseases, Beijing An Zhen Hospital, Capital Medical University, Beijing Institute of Heart, Lung and Blood Vessel Diseases, Beijing, 100029 China; 3 Department of Cardiology, Beijing An Zhen Hospital, Capital Medical University, Beijing, 100029 China; 4 Otolaryngological Department of Beijing An Zhen Hospital, Capital Medical University, Beijing, 100029 China

**Keywords:** Coronary artery disease, Obstructive sleep apnea, Angiopoietin-like proteins, Lipid metabolism

## Abstract

**Purpose:**

Hyperlipidemia is the most important early atherosclerosis and coronary artery disease (CAD) indicator. Angiopoietin-like proteins (ANGPTLs) 3, 4, and 8 are lipid dysfunction markers that may be linked to CAD. We investigated whether these circulating ANGPTLs are associated with CAD in patients with obstructive sleep apnea (OSA).

**Methods:**

A total of 327 individuals participated in this study: 221 patients with OSA and CAD, 50 patients with OSA alone, and 56 controls. The Gensini Score was used to assess the severity of CAD. Serum ANGPTL3, ANGPTL4, and ANGPTL8 were measured in all subjects using Human Magnetic Luminex Screening Assay. The independent association between levels of ANGPTLs and CAD was evaluated by multivariate regression analysis.

**Results:**

Serum ANGPTL3 levels were significantly higher in patients suffering from OSA and CAD compared with patients having OSA alone (46.97 ± 13.89 vs 38.25 ± 15.94 ng/ml, *P* < 0.001). Univariate analysis demonstrated that ANGPTL3 was a risk factor for CAD (OR = 1.72/10 ng ANGPTL3, 95% CI, 1.29–2.28, *P* < 0.001). In addition, multivariate analysis revealed that ANGPTL3 was independently associated with the presence of CAD (OR = 1.74/10 ng ANGPTL3, 95% CI, 1.29–2.35, *P* < 0.001) even after adjusting for cofounding factors. Furthermore, circulating ANGPTL3 levels were positively associated with triglyceride (*r* = 0.16, *P* = 0.01) and total cholesterol (*r* = 0.14, *P* = 0.02) levels, while ANGPTL3 levels had no significant correlation with the severity of CAD. No significant associations were found between the levels of ANGPTL4 and ANGPTL8 and CAD even after adjusting for established risk factors.

**Conclusion:**

Elevated levels of ANGPTL3 were independently associated with a higher likelihood of CAD in patients with OSA. It may be a novel biomarker for OSA patients at high risk of developing cardiovascular diseases.

## Introduction

Obstructive sleep apnea (OSA) is a chronic somnipathy mainly manifesting as repeated apnea, hypopnea, arousal, intermittent hypoxemia (IH), and hypercapnia during sleep [1, 2]. OSA is identified as an independent risk factor for cardiovascular events, including coronary artery disease (CAD), hypertension, strokes, and atherosclerosis [3]. The incidence of CAD in OSA patients is about 20 to 30%, and the incidence of OSA in patients with acute coronary syndrome is as high as 69% [4]. Because cardiovascular disease is among the major causes of death worldwide [5], it is necessary to assess cardiovascular comorbidities in patients with OSA.

Angiopoietin-like proteins (ANGPTLs) comprise a family of secreted proteins, eight members that have a variety of metabolic functions such as insulin resistance, dyslipidemia, and oxidative stress [6]. ANGPTL3, ANGPTL4, and ANGPTL8 are associated with the regulation of lipid metabolism, which is essential for the development of CAD [7].

To date, however, no study has investigated the relationship between circulating ANGPTL3, ANGPTL4, and ANGPTL8 levels and the development and progression of CAD in patients with OSA. We hypothesized that ANGPTLs may be a novel biomarker for patients with OSA at high risk for cardiovascular disease. Therefore, the present study was aimed at investigating the possible role of ANGPTLs in predicting the risk of CAD in patients with OSA.

## Methods

### Patients

**Fig. 1:**
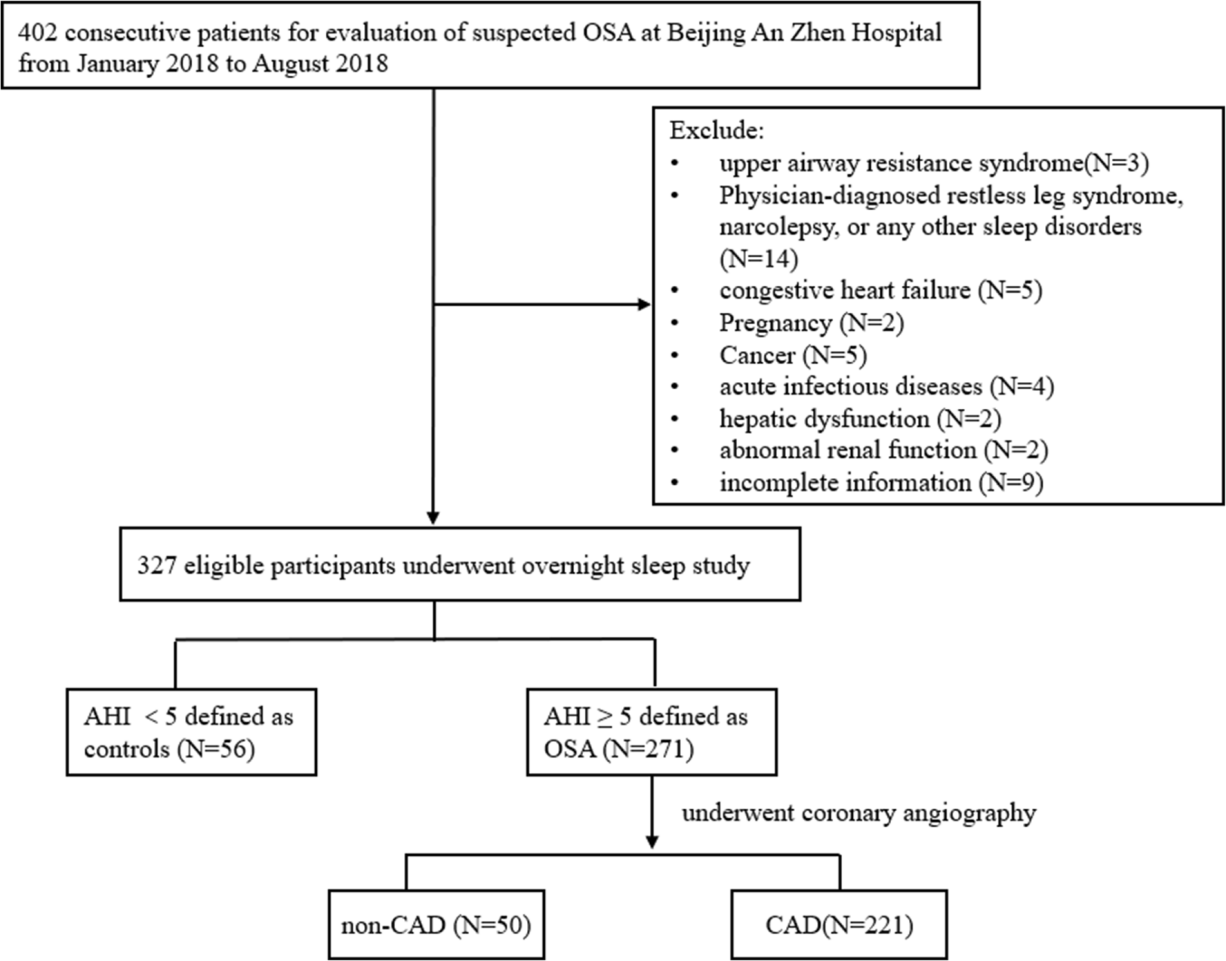
Study flow. About 327 participants were consecutively enrolled including 221 CAD combined OSA patients, 50 non-CAD OSA patients, and 56 non-OSA controls. Abbreviations: OSA obstructive sleep apnea, AHI apnea-hypopnea index, CAD, coronary artery disease

All consecutive patients with suspected OSA, who admitted to the Beijing An Zhen Hospital for polysomnography (PSG) from January 2018 to August 2018, were included in this study. OSA patients were diagnosed on the basis of American Academy of Sleep Medicine Guidelines for an apnea-hypopnea index (AHI) ≥ 5 per hour [8]. Exclusion criteria were other sleep disorders (including restless legs syndrome, narcolepsy), upper airway resistance syndrome, acute infectious disease, cancer, congestive heart failure, renal disease, and hepatic disease. All eligible patients diagnosed with OSA who underwent coronary angiography were classified as patients with or without CAD. CAD was defined as stenosis ≥ 50% of the left main coronary artery or stenosis ≥ 70% of a major epicardial vessel (left anterior descending artery, left circumflex artery, or right coronary artery) [9]. A final total of 327 participants were consecutively enrolled, including 271 patients with OSA and 56 non-OSA controls. According to the diagnostic standard, the OSA patients with OSA were divided into two groups: non-CAD (*n* = 50) and CAD (*n* = 221). The study design is described in detail in Fig. 1.

Demographic data were collected: age, gender, body mass index (BMI), medical history (previously diagnosed hypertension, hyperlipidemia, diabetes mellitus), and current medications. The study has been authorized and registered by Medical Ethics Committee of Beijing An Zhen Hospital (2017005) together with China Clinical Trial Registry (CHICTR-ROC-17011027). All study patients have written informed consent.

### Evaluation of Coronary Severity

The severity of CAD was evaluated by the Gensini Score [10] and the SYNTAX Score [11, 12]. The SYNTAX Score was calculated using an online calculator (http://www.syntaxscore.com/). All the characteristics of and scores for CAD were assessed by two experienced interventional cardiologists blinded to the patients’ baseline characteristics.

### Measurement of Biochemical Parameters

Morning blood samples were drawn from patients after PSG and a 12-h fasting period and then centrifuged at 2400×*g* for 5 min. Specimens were stored at − 80 °C. The commercially available Luminex assay kit, a magnetic bead-based screening assay (R&D Systems, Minneapolis, MN, USA), was used to measure circulating ANGPTL3, ANGPTL4, and ANGPTL8 levels. In our study, intra-assay and inter-assay coefficients of variation (CV) were < 5% and < 10%, respectively. All samples were performed in duplicate and repeated for a CV > 15%. Based on standard laboratory techniques at Beijing An Zhen Hospital, biochemical data for fasting plasma glucose (FPG) and fasting lipid profile, such as total cholesterol (TC), triglyceride (TG), low-density lipoprotein cholesterol (LDL-C), and high-density lipoprotein cholesterol (HDL-C), were measured.

### Statistical Analysis

All statistical analyses used SPSS version 23.0 (IBM Corp., Armonk, NY, USA). A *P* value < 0.05 was considered significant. Data for continuous variables were presented as the mean ± standard deviation for normally distributed data and as the median (interquartile range) for non-normally distributed data. The independent Student’s *t* tests or Wilcoxon’s tests were used to analyze continuous variables, and Chi-squared tests were used for categorical variables. The association between circulating ANGPTL3, ANGPTL4, and ANGPTL8 levels and CAD was determined by multivariate logistic regression analysis. Spearman’s or Pearson’s correlation was used to determine the association between ANGPTLs and the severity of CAD as assessed by the Gensini and SYNTAX Scores. Receiver operating characteristic (ROC) curve analysis was performed to assess ANGPTL level in predicting CAD, and the optimal value was determined depending on the Youden Index.

## Results

### Physical and Clinical Characteristics of Study Subjects

**Fig. 2:**
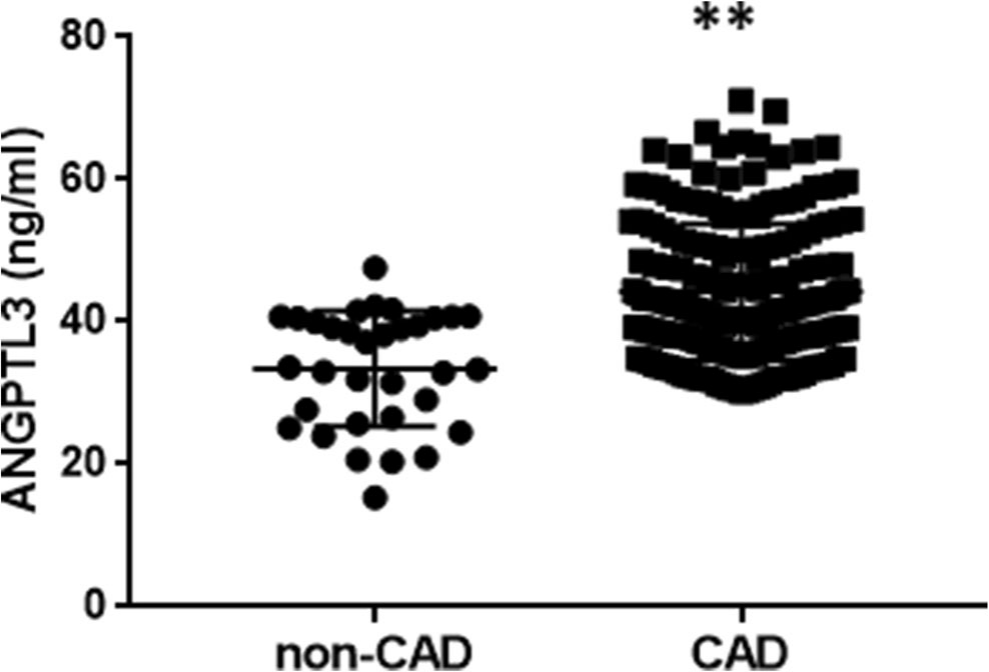
Circulating ANGPTL3 levels were higher in patients with CAD compared with controls. The comparison of the concentration of ANGPTL3 in CAD and non-CAD patients is shown in a dot plot. The concentration in the CAD group is 46.97 ± 13.89 ng/ml, and the concentration in the non-CAD group is 38.25 ± 15.94 ng/ml. Data are expressed as the mean ± standard deviation. Abbreviations: CAD coronary artery disease, ANGPTL3 angiopoietin-like protein 3 ** *P* < 0.001

All subjects’ physical and clinical features are listed in Table 1. Patients with CAD had significantly lower HDL-C levels (*P* < 0.05) and diastolic blood pressure (*P* < 0.05) compared with non-CAD patients. There were no significant differences in age (*P* = 0.612), gender (*P* = 0.598), TG (*P* = 0.091), TC (*P* = 0.703), LDL-C (*P* = 0.771), systolic blood pressure (*P* = 0.133), FPG level (*P* = 0.433), high-sensitivity C-reactive protein (*P* = 0.319), or statin therapy (*P* = 0.972) among three groups. As shown in Fig. 2, the circulating ANGPTL3 level in the CAD group was significantly higher than in the non-CAD group (46.97 ± 13.89 ng/ml vs 38.25 ± 15.94 ng/ml, respectively; *P* < 0.001), while no significant differences were found in ANGPTL4 and ANGPTL8 levels between the two groups (Table 1).

**Table 1:** Anthropometric and biochemical characteristics of the subjects included in the study

	Controls	OSA		*P* value
		non-CAD	CAD	
*N*	56	50	221	
Male (*n*, %)	44 (78.57%)	42 (84.00%)	186 (84.16%)	0.598
Age (years)	58.84 ± 9.44	57.04 ± 11.19	57.46 ± 10.39	0.612
BMI (kg/m^2^)	24.80 ± 3.34	27.71 ± 3.97	26.79 ± 3.34	< 0.001
SBP (mmHg)	128.63 ± 19.22	131.22 ± 16.70	126.00 ± 17.15	0.133
DBP (mmHg)	76.11 ± 12.38	80.98 ± 13.92	75.94 ± 11.93^a^	0.032
FPG (mmol/L)	6.69 ± 2.16	6.15 ± 2.07	6.53 ± 2.24	0.433
TG (mmol/L)	1.29 (0.95–1.83)	1.50 (0.98–1.87)	1.59 (1.13–2.23)	0.091
TC (mmol/L)	4.14 ± 1.14	4.31 ± 1.16	4.24 ± 1.02	0.703
HDL-C (mmol/L)	1.07 (0.90–1.31)	1.17 (0.97–1.30)	1.00 (0.88–1.21)^a^	0.021
LDL-C (mmol/L)	2.42 ± 0.91	2.52 ± 1.00	2.52 ± 0.94	0.771
hs-CRP (mg/ml)	3.77 (0.50–3.17)	1.17 (0.56–2.56)	1.49 (0.60–5.38)	0.319
Smoker (*n*, %)	30 (53.57%)	31 (62.00%)	120 (54.55%)	0.600
Drinker (*n*, %)	19 (33.93%)	19 (38.00%)	84 (38.01%)	0.848
Statin therapy (*n*, %)	-	13 (26.00%)	58 (26.24%)	0.972
ANGPTL3 (ng/ml)	35.04 ± 18.18	38.25 ± 15.94	46.97 ± 13.89^a^*	< 0.001
ANGPTL4 (ng/ml)	158.45 ± 79.31	148.89 ± 61.38	151.84 ± 70.69	0.199
ANGPTL8 (pg/ml)	741.29 ± 203.12	721.87 ± 295.48	735.15 ± 307.96	0.939

Results are expressed as mean ± standard deviation, median (interquartile range), or *n* (%). Differences between groups were analyzed by the independent Student’s *t* test, χ2 text, or Wilcoxon’s test

*OSA* obstructive sleep apnea, *CAD* coronary artery disease, *BMI* body mass index, *SBP* systolic blood pressure, *DBP* diastolic blood pressure, *FPG* fasting plasma glucose, *TG* triglycerides, *TC* total cholesterol, *LDL-C* low-density lipoprotein cholesterol, *HDL-C* high-density lipoprotein cholesterol, *hs-CRP* high sensitive C reaction protein

*P < 0.001

^a^Statistical difference from non-CAD *P* < 0.05

### Association Between Circulating ANGPTL3, ANGPTL4, and ANGPTL8 Levels and CAD

The relationship between ANGPTL3, ANGPTL4, and ANGPTL8 levels and the risk of CAD were further explored using different logistic regression models (Table 2). Univariate analysis demonstrated that ANGPTL3 was a risk factor for CAD (OR = 1.72/10 ng ANGPTL3, 95% CI, 1.29–2.28; *P* < 0.001). After the adjustment for confounding factors, patients with a higher circulating ANGPTL3 level had a higher OR for CAD (OR = 1.74/10 ng ANGPTL3, 95% CI, 1.29–2.35; *P* < 0.001), indicating that ANGPTL3 level is an independent risk factor for CAD. In contrast, no significant associations were found between the levels of ANGPTL4 and ANGPTL8 and CAD even after adjusting for established risk factors.

**Table 2:** Multivariate logistic regression analyses of circulating ANGPTL3, ANGPTL4, and ANGPTL8 levels and CAD

	Unadjusted		Model 1		Model 2	
	OR (95%CI)	*P* value	OR (95%CI)	*P* value	OR (95%CI)	*P* value
ANGPTL3(per 10 ng/ml increase)	1.72 (1.29, 2.28)	< 0.001**	1.78 (1.33, 2.37)	< 0.001**	1.74^a^ (1.29, 2.35)	< 0.001**
ANGPTL4	1.001 (0.996,1.005)	0.784	1.002 (0.997,1.007)	0.475	1.002 (0.997,1.008)	0.362
ANGPTL8	1.000 (0.999,1.001)	0.781	1.000 (0.999,1.001)	0.758	1.000 (0.999,1.001)	0.696

Model 1: adjusted for age, sex, and BMI. Model 2: adjusted for Model 1+ FPG, SBP, DBP, TG, TC, HDL-C, LDL-C, smoke, drink, and statin therapy

*OR* odds ratio, *ANGPTL3* angiopoietin-like protein 3, *ANGPTL4* angiopoietin-like protein 4, *ANGPTL8* angiopoietin-like protein 8, *CAD* coronary artery disease

**P < 0.001

^a^Adjusted for Model 2 + ANGPTL4 + ANGPTL8

ANGPTL3 was an independent risk factor for CAD performed by logistic regression analysis. The degree of association was expressed by OR value

**Fig. 3:**
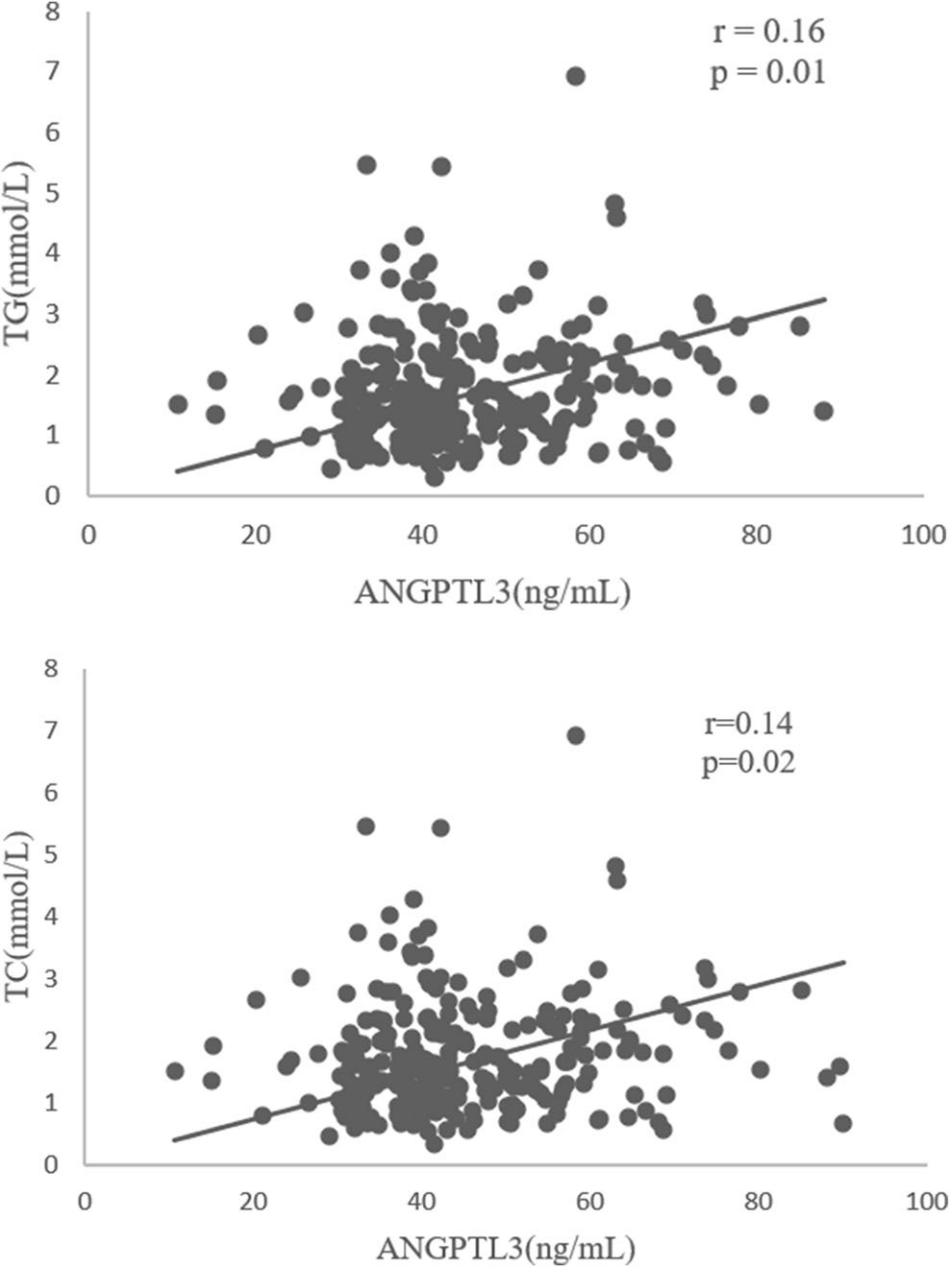
ANGPTL3 was positively correlated with TG and TC. Correlation between TG/TC and ANGPTL3. There was significant positive correlation between TG/TC and ANGPTL3. ANGPTL3 angiopoietin-like protein 3, TG triglycerides, TC total cholesterol

The relationship between ANGPTL3 and the severity of CAD was also determined (Table 3). The ANGPTL3 level positively correlated with the levels of TG (*r* = 0.16, *P* = 0.01) and TC (*r* = 0.14, *P* = 0.019) (Fig. 3), while the circulating ANGPTL3 level had no positive correlation with the Gensini Score (*r* = 0.11, *P* = 0.104) or SYNTAX Score (*r* = 0.09, *P* = 0.20).

**Table 3:** Correlations of ANGPTL3 with clinical parameters

Parameter	Correlation coefficient	*P* value
BMI (kg/m^2^)	0.06	0.329
SBP (mmHg)	− 0.09	0.149
DBP (mmHg)	− 0.10	0.103
TG (mmol/L)^b^	0.16	0.010a
TC (mmol/L)	0.14	0.019
LDL-C (mmol/L)	0.09	0.120
HDL-C (mmol/L)^b^	0.05	0.388
FPG (mmol/L)	− 0.05	0.542
hs-CRP (mg/ml)	0.07	0.243
Gensini Score	0.11	0.104
SYNTAX Score	0.09	0.200

ANGPTL3 was positively correlated with TG and TC. Spearman’s correlation analysis was used for non-normally distributed variables, and Pearson’s correlation analysis was used for normally distributed variables

*BMI* body mass index, *SBP* systolic blood pressure, *DBP* diastolic blood pressure, *TG* triglycerides, *TC* total cholesterol, *LDL-C* low-density lipoprotein cholesterol, *HDL-C* high-density lipoprotein cholesterol, *FPG* fasting plasma glucose, *hs-CRP* high sensitive C reaction protein

*P < 0.05

^a^Significant correlation as assessed by Spearman’s correlation method

^b^Non-normally distributed variables

### ROC Curve Analysis for ANGPTL3

**Fig. 4:**
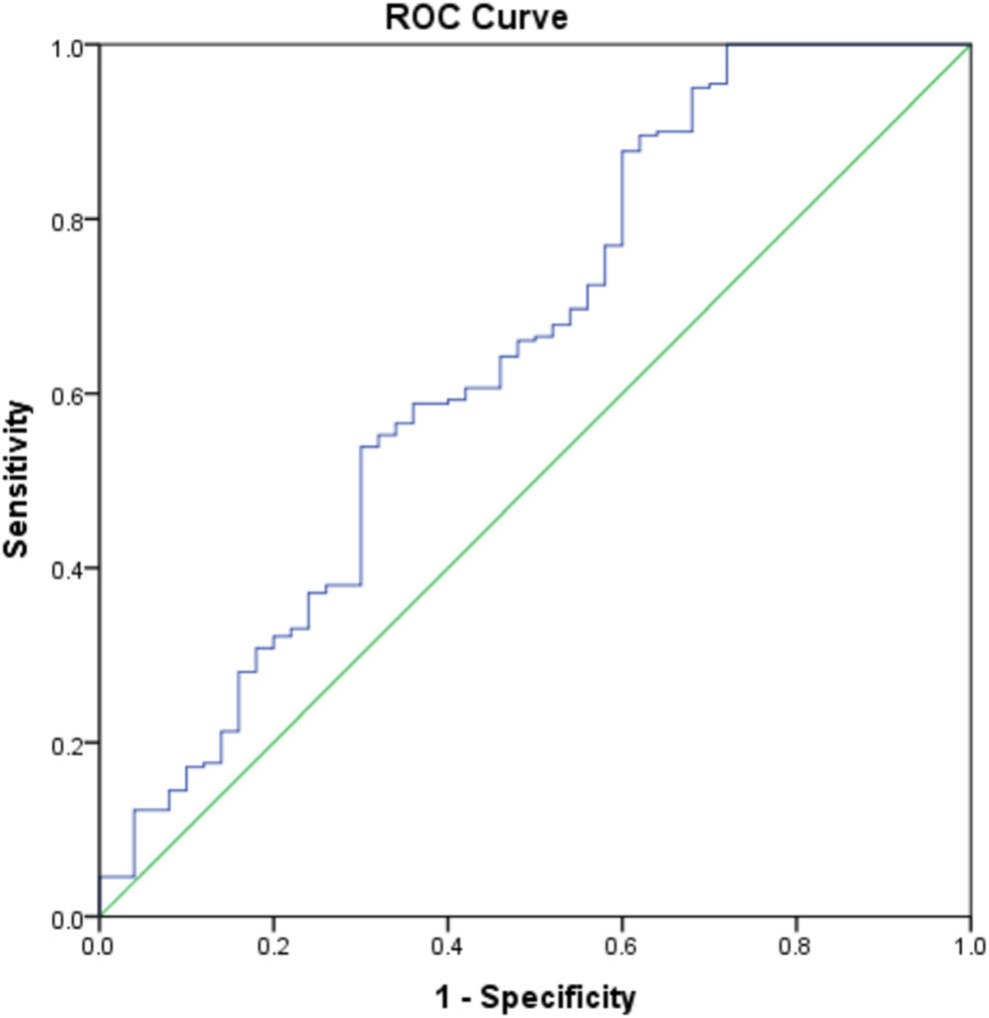
Receiver operating characteristic (ROC) analysis. ROC for predicting CAD using ANGPTL3. The area under the curve (AUC) for the plasma ANGPTL3 levels was 0.65, *P* = 0.001

We performed receiver operating characteristic curve analysis to evaluate the diagnostic performance of ANGPTL3 in discriminating CAD. The area under the curve (AUC) for detecting CAD based on ANGPTL3 was 0.65 (optimal cutoff value, 29.68 ng/ml, sensitivity, 100%; specificity, 28%; Youden Index, 0.28; *P* = 0.001) (Fig. 4).

## Discussion

In this research, we demonstrated that the ANGPTL3 levels of CAD patients were clearly higher compared with non-CAD subjects in OSA patients. We first addressed the relationship between ANGPTL3 level and CAD in patients with OSA. The results showed that ANGPTL3 was an independent predictor of CAD risk. Further analysis indicated that ANGPTL3 level had a significant correlation with lipid profile abnormality.

ANGPTL3, ANGPTL4, and ANGPTL8 are important factors in the regulation of the metabolism of lipids and lipoproteins, providing new hope for the treatment of hyperlipidemia [7, 13]. Lipid dysfunction is believed to be the initial abnormality in the development of early atherosclerosis and coronary artery disease. This indicated that ANGPTL3, ANGPTL4, and ANGPTL8 play important role in cardiovascular disease through regulation of lipid metabolism. It is reported that ANGPTL3 deficiency protects against CAD. Heterozygous carriers of ANGPTL3 loss-of-function mutations have a 34% reduction in the odds of developing CAD. Individuals in the lowest tertile of circulating ANGPTL3 concentration, compared with the highest, had reduced odds of myocardial infarction [14]. ANGPTL3 single-nucleotide polymorphisms and their haplotypes are associated with the severity of coronary artery atherosclerosis and the risk of CAD, as assessed by angiography [15]. In addition, the fasting serum ANGPTL3 level positively correlates with the aortic augmentation index value among patients with CAD [16]. Our study also found that increased circulating ANGPTL3 was associated with a high risk of CAD in patients with OSA. The Gensini and SYNTAX Scores are valid and reliable systems that assess the extent and severity of CAD. Circulating ANGPTL8 level was an independent risk factor for CAD and was found to be positively associated with the Gensini Score in non-diabetic patients [17]. However, our study found no significant correlation between serum ANGPTL8 level and CAD. The reason for this discrepancy might be that the study population differs between the two studies. This study included patients with diabetes. It is reported that ANGPTL8 concentrations were further reduced up to 70% in obese participants with diabetes [18]. Another study indicated that ANGPTL8 concentration exhibited no significant difference between CAD group and non-CAD group [19] and ANGPTL8 levels were not a factor for CAD in the multivariate analysis [20], which is consistent with our results. No significant association was found between ANGPTL4 level and angiographically characterized coronary atherosclerosis and severity of CAD [21, 22]. Consistent with previous reports, our study did not find a significant association between ANGPTL4 and CAD.

Compared with the general population, patients with OSA have a higher risk of cardiovascular disease [23], partly because of atherogenic dyslipidemia. Chronic intermittent hypoxia (CIH) is the main factor in the pathogenesis of OSA. CIH induced an 80% increase in ANGPTL4 gene expression with a corresponding increase in protein level [24]. Another study reported that ANGPTL4 was induced by hypoxia in isolated cardiomyocytes in vitro via the hypoxia-inducible factor 1 regulatory pathway [25]. Hypoxia increased the level of adipose ANGPTL4. By inhibiting lipoprotein lipase, fasting levels of plasma TG and very low-density lipoprotein cholesterol were increased, thereby increasing the size of atherosclerotic plaques. This effect was eliminated by antibodies [26]. Consistent with the results of animal experiments, clinical studies have also found that ANGPTL4 and ANGPTL8 levels were increased in subjects with OSA and positively correlated with TG [27, 28]. In our study, we also investigated the relationship between ANGPTL3 and lipids, and we found that the circulating ANGPTL3 level was positively correlated with TG. This finding suggests that ANGPTL3 regulates lipid metabolism and may partially explain the high CAD risk observed in patients with OSA. However, there is no correlation with LDL-C, which may be related to the treatment with statins of patients.

Despite the fact that LDL-C is causal in the development of atherogenesis and CAD, new options are required to control high TG level. The magnitude of the contribution of TG level to CAD risk is evident from both long-term prospective studies [29] and genetic analyses [30]. Genetic observations strongly support the utility of developing new ANGPTL3 inhibitors to reduce TG level and the incidence of cardiovascular disease. Evinacumab is a fully human monoclonal antibody directed toward ANGPTL3 [31] that can reduce the TG level in healthy people and patients with homozygous familial hypercholesterolemia [32]. Our study revealed that ANGPTL3 is an independent risk factor for CAD and positively correlated with lipid levels in patients with OSA. These observations are quite promising and have led us to consider whether evinacumab could be used to control blood lipids in patients with OSA and thus greatly reduce their cardiovascular risk. This study has some limitations. First, the relatively small sample size might be statistically insufficient to validate our results. Second, the cross-sectional evidence requires confirmation from a prospective cohort study. Finally, a subset of the study participants were taking medication, which may have affected the levels of ANGPTL3, ANGPTL4, and ANGPTL8 in this study.

## Conclusions

This study indicates that the circulating level of ANGPTL3 was increased in patients with OSA and CAD compared with patients with OSA alone. Moreover, ANGPTL3 level was independently correlated with the presence of CAD in patients with OSA. These observations may explain, in part, the high CAD risk in subjects with OSA. Therefore, an elevated ANGPTL3 level may be a significant clinical target in the diagnosis and effective treatment of CAD in patients with OSA.

## Data Availability

Availability of data and material has been described in the manuscript. They are freely available to any scientist who wishes to use them without breaching participant confidentiality.
